# Difficult-to-treat and severe asthma in general practice: delivery and evaluation of an educational program

**DOI:** 10.1186/s12875-019-0991-y

**Published:** 2019-07-13

**Authors:** Isla Hains, Josh Meyers, Kirsten Sterling, Jeannie Yoo, Helen Reddel, Clare Weston

**Affiliations:** 1 NPS MedicineWise, PO Box 1147, Strawberry Hills, NSW 2012 Australia; 2 Woolcock Institute of Medical Research, The University of Sydney, Sydney, NSW 2006 Australia

**Keywords:** Asthma, Severe asthma, Difficult-to-treat asthma, Uncontrolled asthma, Evaluation, Medical education, Training, Quality improvement, General practitioner

## Abstract

**Background:**

Asthma, a common yet complex airway disorder affecting about 11% of Australians, is well-controlled in only 54% of people with asthma. Those with difficult-to-treat and severe asthma are more likely to experience recurrent and potentially life-threatening exacerbations. It is therefore important that GPs can initiate a systematic approach for the management of patients with difficult-to-treat asthma to identify those whose condition may improve by addressing contributory factors and those who require specialist input. We therefore aimed to develop and deliver an educational program for GPs on the systematic management of patients with difficult-to-treat and severe asthma and evaluate the effectiveness of this program.

**Methods:**

We developed an educational program on the management of difficult-to-treat and severe asthma in primary care that was delivered to GPs and other health professionals between January and June 2018. We evaluated the effectiveness of the program using a retrospective pre-test with post-survey, administered to GPs directly after program participation.

**Results:**

Over 1000 general practice health professionals participated in the educational program, including 890 GPs of whom 226 (25%) completed the survey. Following program participation, a greater proportion of GPs identified factors they would assess in managing a patient with poor asthma control, particularly for considering the risk of future adverse outcomes (+ 51%), changes in lifestyle (+ 38%), and self-management strategies (+ 35%). GPs indicated a greater awareness of the biologic therapies that specialists could consider prescribing to their patients with severe asthma (+ 75%), of the requirements for a patient to be prescribed a biologic therapy (+ 73%) and that patients with different phenotypic characteristics can respond differently to standard therapy (+ 67%). The proportion of GPs who would refer appropriate patients to a specialist also significantly increased.

**Conclusions:**

This study suggests that an evidence-based educational program can improve GP knowledge, confidence and intended practice in managing patients with difficult-to-treat and severe asthma.

**Electronic supplementary material:**

The online version of this article (10.1186/s12875-019-0991-y) contains supplementary material, which is available to authorized users.

## Background

Asthma is a common yet complex airway disorder that affects approximately 11% of the Australian population [1]. A 2012 Australian survey found that asthma was well-controlled in only 54% of people with asthma [2]. The management of adults with asthma that remains uncontrolled despite treatment with high-dose therapies is challenging for both clinicians and patients [3]. Although most people’s asthma can be effectively treated by general practitioners (GPs) with available medicines, a significant population has uncontrolled asthma despite high-dose treatment. These patients are categorised as having difficult-to-treat asthma [4]. Poor asthma control in these patients is most commonly due to factors other than asthma itself — including poor adherence to treatment or incorrect inhaler technique [5]. This is not the case however for those with true severe asthma, whose asthma remains uncontrolled or fails to improve despite treatment optimisation, confirmation of the diagnosis and treatment of confounders [6]. Severe asthma is a subset of difficult-to-treat asthma and these definitions are commonly misunderstood by healthcare professionals [3, 4]. Patients with severe asthma, who make up 3 to 10% of the population of adults with asthma, are at increased risk for medication-related side-effects, and are more likely to experience recurrent and potentially life-threatening exacerbations [7].

Unlike other disease conditions, asthma severity is assessed retrospectively based on the level of treatment required to achieve good asthma control. It can therefore only be assessed when a patient has been on inhaled anti-inflammatory therapy, commonly called a ‘preventer’ (or controller), for several months [6, 8]. Asthma control, however, is described in terms of both recent symptom control and future risk of adverse outcomes [5]. Asthma control and severity are not synonymous and should not be used interchangeably [5, 9].

Recent advances in the understanding of the pathophysiology of asthma, and recognition of its phenotypic heterogeneity, have prompted the development of new biologic medicines for the treatment of severe asthma [10]. However, GPs may be unfamiliar with this relatively new group of medicines and their availability. These medicines can offer important clinical benefits to selected patients whose asthma is relatively refractory to conventional treatment [11]. Biologic therapies are expensive, and within Australia government subsidies to help with medicine costs via the Pharmaceutical Benefits Scheme (PBS) are only available for patients who meet strict criteria [12–14]. It is therefore important that GPs can initiate a systematic approach for the management of patients with difficult-to-treat asthma to identify those who may improve by addressing contributory factors and identify those who require specialist input [5].

NPS MedicineWise is an independent, not-for-profit and evidence-based organisation that works to improve the way medicines, medical tests and health technologies are prescribed and used in Australia. We offer a visiting education service for health professionals and deliver over 25,000 face-to-face visits each year to GPs in their practices. Visits are delivered by a national team of educational visitors either one-to-one or in small group meetings, supporting achievement of quality use of medicines and other health technologies through evidence-based academic detailing.

The aims of this study were to develop and deliver an educational visiting program to GPs on the systematic guidelines-based management of patients with difficult-to-treat and severe asthma and to evaluate the changes in GP knowledge and intended practice as a result of this program.

## Methods

### Participants and setting

We designed an educational program that focused on the management of difficult-to-treat and severe asthma in primary care. This program was delivered by NPS MedicineWise educational visitors to GPs and their interested practice staff (e.g., practice nurses, Aboriginal health workers) at Australian general practices in the states of New South Wales, Queensland and Victoria, between January 2018 and June 2018.

Delivery of the program was targeted to reach GPs in areas where there were higher rates of uncontrolled asthma, as indicated by higher asthma-related healthcare utilisation. The target delivery areas were identified using:

- analysis of MedicineInsight data to identify areas with high rates of asthma-related GP encounters. MedicineInsight is an Australian general practice data program developed and managed by NPS MedicineWise, with funding support from the Australian Government Department of Health [15]. It is a large-scale national general practice data program that extracts and collates longitudinal, de-identified patient health records from selected clinical information systems. These data are used for research, to inform health care decision making and policy [16–18].
- Australian Atlas of Healthcare Variation to identify areas with high rates of hospital admissions for asthma among people aged 20–44 years (standardised for population density) [19].

For visits in this program, we prioritised areas with both high hospitalisation rates and high rates of asthma-related GP encounters in regional and metropolitan areas.

### Education program design

We designed the educational program based on academic detailing principles, which have been shown to be effective in influencing health professionals’ knowledge and practice [20, 21]. The program content was informed by a review of the literature, key informant interviews with practising GPs, and an extensive stakeholder consultation process that included engagement with national asthma organisations as well as several expert respiratory physicians and GPs.

This process enabled the development of evidence-based program objectives, key education messages and a framework for delivering the educational program to small groups of GPs and allied health staff in their practices (Table 1). The program was delivered through a facilitated face-to-face 1-h meeting which included a discussion of the key messages (see Table 1) as well as discussion of how to differentiate between difficult-to-treat and severe asthma, modifiable factors that may contribute to poor symptom control, a treatment and referral pathway algorithm and a summary of currently available biologic therapies.

**Table 1:** Program objectives and key messages

*Program objectives*	Increase GP knowledge about difficult-to-treat asthma and its subtypes
	Increase the proportion of GPs who can identify patients with poorly controlled asthma
	Increase the proportion of GPs who can identify severe asthma cases within the broader difficult-to-treat asthma group
	Increase awareness of benefits of referral to respiratory specialists for patients with severe, high risk or difficult-to-treat asthma
*Key messages*	Identify patients with uncontrolled or difficult-to-treat asthma
	Assess and manage factors contributing to poor asthma control (to differentiate between difficult-to-treat and severe asthma)
	Consider referral to a specialist respiratory physician for people with severe, high-risk or difficult-to-treat asthma.

Resources to support the educational program were developed and pilot tested with GPs. These resources included:

- a GP handout – a paper resource (see Additional file 1) with detailed program content designed to support and complement the discussion between the GP and educational visitor. This was left with GPs for reference. Two case studies were included in this resource to facilitate peer group discussion of a systematic approach to managing patients with difficult-to-treat asthma
- a discussion guide for the educational visitors to help facilitate and guide discussions within the meeting.

Ten experienced educational visitors were trained to deliver the educational program through background reading and a half-day training workshop including role playing and case discussion. Throughout delivery of the educational program, the educational visitors received ongoing clinical support from the program team via an online discussion forum and regular teleconferences.

The educational program was accredited as continuing professional development with the peak Australian general practice colleges.

### Evaluation

#### Data collection

We designed a retrospective pre-test (RPT) with post-survey to measure changes in GP knowledge, attitudes and practice following the educational program [22–27]. The RPT approach asks GPs to assess what they know or would do from two viewpoints – BEFORE and AFTER participating in the educational program. The questionnaire (Additional file 2) included questions that related to GP understanding of poorly controlled asthma, how GPs approach interactions with patients with poorly controlled asthma, and their knowledge and confidence in managing patients with difficult-to-treat asthma.^1^ The questionnaire was developed with GP and respiratory specialist input.

The questionnaire was initially administered via an online survey three weeks after the educational visit with up to two electronic reminders. After the first 443 GP visits the response rate was 10%, raising concerns about the representativeness of data on changes in GP knowledge, attitudes and practice. For subsequent visits, GPs were provided with a paper copy of the questionnaire at completion of the educational visit, and they received an email reminder one week after the visit with a link to an electronic version of the survey. To maximise the response rate, GPs were provided with the option to return the survey at a time most convenient for them (i.e., directly after the visit, or later by fax, mail or online).

#### Ethics and consent

Ethics approval for the questionnaire, and its amendment following the change to data collection, was obtained from the Royal Australian College of General Practitioners National Research and Evaluation Ethics Committee (NREEC 17–018). GPs were provided with an information sheet that explained the study and highlighted that survey completion implied consent. Written consent to participate was therefore not obtained from participating GPs.

#### Data analysis

We analysed the survey data using SPSS statistical software (IBM, version 23). Descriptive statistics were calculated for each survey item and McNemar’s test was used for paired comparisons [28]. No adjustment was made for multiple comparisons.

We also compared the survey data for the GPs who received the online survey three weeks after the educational visit and those GPs who received the paper survey directly after the educational visit. Most responses were consistent between both groups.

## Results

A total of 1,080 general practice health professionals, including 890 GPs, participated in the educational program, in a total of 185 visits. Overall 226 GPs (25%) completed the survey, though not all GPs answered every question. Of these, 48% (*n* = 108) were male and 52% (*n =* 115) were female. GP survey respondents had practised for an average of 15.7 years, saw an average of 113 patients per week and a median of 10 patients with asthma per week. Overall, most GPs were entirely satisfied with the educational visit (96%, *n =* 214).

### Understanding of asthma definitions

GP understanding of the different asthma terms was mixed following the educational visit (Table 2). GPs could select more than one asthma definition per statement though most GPs appropriately identified uncontrolled asthma (76%, *n =* 168) and difficult-to-treat asthma (69%, *n =* 154). However, 43% of GPs selected severe asthma for statement 2 which is most appropriately described as difficult-to-treat asthma. Of those selecting difficult-to-treat asthma for this statement, 69% chose this as their sole option and 17% additionally selected severe asthma (data not shown), which is not the most correct response. Similarly, for statement 3, which describes a patient with severe asthma, 56% of GPs (*n =* 124) appropriately identified this, however 45% also selected difficult-to-treat asthma (*n =* 100). Of those selecting severe asthma, 70% chose this as their sole option and 10% also selected difficult-to-treat asthma (data not shown).

**Table 2:** GP selection of asthma definitions for each survey statement after the educational visit (*N =* 222)

Statement^a^ *(Desired response)*	Uncontrolled asthma, n (%)	Difficult-to-treat asthma, n (%)	Severe asthma, n (%)
1. Asthma that includes at least one of the following: poor symptom control, frequent severe exacerbations, serious exacerbations and/or airflow limitation ***(Uncontrolled asthma)***	168 (76%)	61 (28%)	86 (39%)
2. Asthma that is uncontrolled despite high-dose ICS/LABA and/or oral corticosteroids, or that requires such treatment to remain well controlled ***(Difficult-to-treat asthma)***	41 (19%)	154 (69%)	96 (43%)
3. Asthma that is uncontrolled despite high-dose ICS/LABA and/or oral corticosteroids and does not improve following appropriate diagnosis, optimisation of inhaled treatment and/or treatment of confounders ***(Severe asthma)***	61 (28%)	100 (45%)	124 (56%)

Abbreviations: *ICS* Inhaled corticosteroid, *LABA* Long-acting beta_2_ agonist

^a^GPs could select more than one asthma definition per statement

### Asthma management

After participating in the educational visit, more GPs identified factors associated with poor asthma control (Table 3). The largest increase was seen in the proportion of GPs who would consider the risk of future adverse outcomes when managing a patient with poorly controlled asthma (+ 51%). This was followed by changes in lifestyle (+ 38%), and self-management strategies (+ 35%). Practice appeared high before the program for some topics where more than 85% of GP respondents reported already considering trigger factors, medicine adherence and adequate trial of preventer medicines. So minimal change was seen for these factors.

**Table 3:** Factors considered by GPs when managing patients with poorly controlled asthma (*N =* 181)

Factors	BEFORE, n (%)	NOW, n (%)	Difference, *p* value
Risk of future adverse outcomes	65 (36%)	158 (87%)	+ 51%; *p* ≤ 0.001
Changes in lifestyle (exercise, weight, etc)	99 (55%)	168 (93%)	+ 38%; *p* ≤ 0.001
Self-management strategies	100 (55%)	163 (90%)	+ 35%; *p* ≤ 0.001
Confirmation of diagnosis with spirometry	120 (66%)	162 (90%)	+ 24%; *p* ≤ 0.001
Use of a written action plan	122 (67%)	160 (88%)	+ 21%; *p* ≤ 0.001
Comorbidities	133 (74%)	167 (92%)	+ 18%; *p* ≤ 0.001
Inhaler technique	150 (83%)	167 (92%)	+ 9%; *p* ≤ 0.01
Adequate trial of preventer medicines	153 (85%)	161 (89%)	No difference
Medicine adherence	162 (90%)	169 (93%)	No difference
Trigger factors	162 (90%)	165 (91%)	No difference

Overall GP knowledge and confidence about managing patients with difficult-to-treat and severe asthma was reported as improving after participating in the educational visit (Table 4). For each survey statement, there was a significant difference (*p* ≤ 0.001) in the proportion of GPs who agreed/strongly agreed with each statement before and after participating in the educational visit.

**Table 4:** Proportion of GPs agreeing with statements about management of patients with difficult-to-treat and severe asthma

Statement	BEFORE, n (%)	NOW, n (%)	Difference, *p* value
I have a good understanding of which patients would benefit from timely referral to a respiratory specialist	125 (66%)	214 (97%)	+ 31%; *p* ≤ 0.001
I understand that different patients with asthma may have different phenotypic characteristics that can respond differently to standard therapy	57 (30%)	212 (97%)	+ 67%; *p* ≤ 0.001
I am aware of available biologic therapies that respiratory specialists can consider prescribing to my patients with severe asthma	39 (21%)	210 (96%)	+ 75%; *p* ≤ 0.001
I have a good understanding of the role of GPs and specialists in collaboratively managing a patient with severe asthma who requires biologic therapy	84 (44%)	202 (91%)	+ 47%; *p* ≤ 0.001
I would feel confident managing a patient I have identified as having difficult-to-treat asthma	90 (49%)	195 (88%)	+ 39%; *p* ≤ 0.001
I understand the PBS requirements for a patient to be prescribed a biologic therapy for severe asthma	24 (13%)	191 (86%)	+ 73%; *p* ≤ 0.001

The greatest knowledge increases related to GP awareness of the biologic therapies that specialists could consider prescribing to their patients with severe asthma (+ 75%), of PBS requirements for a patient to be prescribed a biologic therapy (+ 73%), and knowing that patients with different phenotypic characteristics can respond differently to standard therapy (+ 67%).

The proportion of GPs who stated they would be confident in managing a patient they identified as having difficult-to-treat asthma also increased (+ 39%), as did the proportion of GPs who felt they had a good understanding of which patients would benefit from referral to a respiratory specialist (+ 31%).

### Specialist referral for asthma

Following participation in the educational program, the proportion of GPs who would refer appropriate patients to a specialist significantly increased (Table 5). The greatest increase was observed in the proportion of GPs who would refer their patient to a specialist when they thought their patient might have severe asthma and could be a candidate for treatment with a biologic therapy. Before the visit, 24% of GPs considered referral in this case, and afterwards 98% would, representing an increase of 75% (*p* ≤ 0.001). In many situations, a large proportion of GPs already appeared to appropriately refer their patients to specialists prior to the education program. However, significantly greater proportions of GPs would consider appropriate referrals after the program, with increases of between 11 and 29% seen (*p* ≤ 0.05).

**Table 5:** Proportion of GPs who consider referring a patient to a respiratory specialist in specific situations (*N =* 179)

Situation	BEFORE, n (%)	NOW, n (%)	Difference, *p* value
I believe the patient may have severe asthma and may be a candidate for treatment with a biologic therapy	42 (24%)	176 (98%)	+ 75%, *p* ≤ 0.001
I suspect the patient has occupational asthma	101 (56%)	152 (85%)	+ 29%, *p* ≤ 0.001
The patient is at high risk of future adverse asthma-related outcomes	109 (61%)	159 (89%)	+ 28%, *p* ≤ 0.001
I have addressed comorbidities I can treat and have not seen an improvement in asthma control	116 (65%)	159 (89%)	+ 24%, *p* ≤ 0.001
The patient requires high-dose inhaled corticosteroids to maintain asthma control despite correct inhaler technique and good adherence	117 (65%)	145 (81%)	+ 16%, *p* ≤ 0.001
I am unsure of the asthma diagnosis (e.g., patients with features of both asthma and COPD^a^)	132 (74%)	151 (84%)	+ 11%, *p* = 0.018
I have been treating the patient for 12 months or more with little to no improvement in symptoms	146 (82%)	158 (88%)	+ 7%, No significant difference

^a^Chronic obstructive pulmonary disease

## Discussion

This educational program sought to improve the assessment and management of patients with difficult-to-treat and severe asthma in Australian primary care. The educational program focused on a systematic approach to difficult-to-treat asthma, including identification, management and referral to specialist care if appropriate. GP knowledge of and confidence in managing patients with difficult-to-treat and severe asthma improved after participating in the educational program. Our evaluation results suggest that, following participation in the program, a greater proportion of GPs felt they better understood the concept of asthma severity and its therapeutic implications, including identification of patients who could benefit from timely referral to a respiratory specialist, and the role of biologic therapy for patients with severe asthma.

The concept of asthma severity is commonly misunderstood in primary care, not surprisingly since this concept has changed substantially over time [9]. While GPs reported that their knowledge of managing patients with difficult-to-treat and severe asthma increased, their understanding of asthma classification was mixed following the educational program, specifically their knowledge of the definitions of asthma severity. This is not surprising given the complexity of assessing asthma severity: defining severity based upon intensity of treatment, and retrospectively applying a severity label after 3–6 months treatment is not a common method for classifying the severity of other diseases. This issue is further confounded by the common use of several severity descriptors for other features such as the intensity and frequency of symptoms, severity of exacerbations (e.g., ‘acute severe asthma’) and degree of obstruction (e.g., ‘severe airflow limitation’) [29]. As frequent and severe symptoms may rapidly become well controlled with optimised ICS therapy for many patients, these features at the time of presentation cannot be considered to reflect underlying severe disease. The distinction between difficult-to-treat and severe asthma also has important therapeutic implications: biologic therapies in Australia are reserved only for those with specific phenotypes of severe asthma who are likely to respond. However, many patients presenting with difficult-to-treat asthma are likely to improve with less costly interventions, such as managing comorbidities. Additional education in these areas might be worth considering for future programs.

Consideration of factors contributing to poor asthma control is an important step in the systematic approach to difficult-to-treat asthma. Self-reported measures of GP awareness of these factors improved following the educational program. This has important clinical implications for improving patient outcomes as recent studies have found that one-third of patients with doctor-diagnosed asthma had no evidence of asthma after objective assessment [30]; almost half of patients with difficult-to-treat asthma were non-adherent to their asthma preventers [31]; and only 31% of patients with asthma demonstrated correct inhaler technique [32]. Despite the clinical significance of comorbidities that can mimic asthma or worsen asthma control, they are commonly under-recognised in difficult-to-treat asthma [33]. Detection of these is an essential component of the systematic evaluation of asthma, as management of comorbidities such as gastro-oesophageal reflux disease, rhinitis, chronic rhinosinusitis and obesity can often be initiated in primary care.

Diagnosis and management of patients with difficult-to-treat and severe asthma requires coordinated efforts between primary and secondary care [34]. It is important therefore that GPs can identify appropriate patients who may benefit from timely referral to specialist care for review and potential consideration of biologic therapy. Our results highlight a low baseline level of awareness of the role of biologic therapy in severe asthma, which may disadvantage those patients who stand to benefit from timely access to these medicines. With the promise of additional therapies for severe asthma, these findings support further education to improve access.

Although these results demonstrate self-reported understanding and intended behaviour rather than long-term practice and outcomes, they do suggest that when GPs who participated in the program are presented with patients whose asthma is poorly controlled they will now, on their own assessment, be better equipped to manage these patients more appropriately.

The RPT design was chosen as the most appropriate method to evaluate this program as it can overcome important biases such as response shift bias. It is viewed as at least equivalent to, or by some authors, as superior to traditional pre and post survey comparisons and is a pragmatic choice when time and/or resources are limited [25, 26, 35]. Potential limitations of the RPT method are the biases that can be introduced into the data, including effort justification, social desirability and recall bias, although the latter is less likely to be an issue in this particular evaluation [36–38]. Additional limitations of the survey relate to data being self-reported, as described above, and the ethics-approved change in the method of data collection, as described in the Methods. This change resulted in an increased survey response rate, with an overall response rate of 25% when the survey was administered at the end of the visit compared with 10% for the initial surveys that were administered by email 3 weeks post-visit. In recent years there has been an overall decline in survey response rates among Australian GPs [39], thought to be partly due to increased workloads and an increasing number of requests to complete surveys [40]. Actual changes in GP behaviour could not be measured. Other study designs would be required to detect longer term changes in GP practice or patient outcomes such as quality of life, asthma exacerbations or changes in asthma hospitalisations, to determine if education programs that focus on complex chronic conditions such as asthma can impact patient outcomes.

## Conclusions

This educational program improved GP knowledge, confidence and intended practice in managing patients with difficult-to-treat and severe asthma. As a result of the educational program, GPs indicated an increase in knowledge and confidence to: identify patients with difficult-to-treat asthma and severe asthma; address modifiable factors that may contribute to poor asthma control; and identify and refer patients who may benefit from timely specialist referral. Overall, this study demonstrates the value of an educational program that focuses on the management of complex clinical areas and highlights the value of ongoing education in these areas.

## Additional files

Additional file 1: The GP handout provided to GPs as part of the educational visit. (PDF 869 kb)

Additional file 2: The RPT questionnaire provided to GPs at the end of the educational visit. (PDF 385 kb)

## Data Availability

The datasets generated and analysed during the current study are not publicly available due to ethical restrictions.

## Abbreviations

COPD: Chronic obstructive pulmonary disease
GP: General practitioner
ICS: Inhaled corticosteroid
LABA: Long-acting beta_2_ agonist
PBS: Pharmaceutical Benefits Scheme
RPT: Retrospective pre-test

## Footnotes

1 The questionnaire also included compulsory questions required for continuing professional development accreditation, however we do not report on these in this paper.
