# Supplementary material for: Difficult-to-treat and severe asthma in general practice: delivery and evaluation of an educational program
Source: BMC Fam Pract. 2019 Jul 13;20:99. doi: 10.1186/s12875-019-0991-y (PMC6626400; doi:10.1186/s12875-019-0991-y)
Supplement: Supplementary file 1 — The GP handout provided to GPs as part of the educational visit. (PDF 869 kb) [file 12875_2019_991_MOESM1_ESM.pdf]

MANAGEMENT OF PEOPLE WITH DIFFICULT-TO-TREAT ASTHMA: A SYSTEMATIC APPROACH

Though there have been significant advances in the treatment of mild-to-moderate asthma, severe asthma that is refractory to standard treatment remains a significant health problem.[1,2]

Assess patients with difficult-to-treat asthma systematically to differentiate between difficult-to-treat and severe asthma (see Figure 1)[3]

- **Difficult-to-treat asthma** is asthma that is uncontrolled despite high-dose ICS/LABA or OCS, or that requires such treatment to remain well controlled.[1]
- **Severe asthma** is a subset of difficult-to-treat asthma and is largely a diagnosis of exclusion, that is, the exclusion of asthma that appears difficult to treat but that markedly improves after appropriate diagnosis and/or treatment of confounders.[2,3]

CASE STUDY 1: Brody presents for a review of his asthma

- 38 years old, asthma since age 8 years
- Spirometry-confirmed asthma at age 25 years

Current history

- Daytime symptoms 4–5 times/week for the last few months
- Needs reliever 3–4 days a week
- Symptoms affect ability to play cricket despite pre-exercise salbutamol
- ED admission last year due to flare-up not responding to salbutamol
- Two courses of OCS in the last year

Past medical history

- Rhinitis

Social

- Ex-smoker – 15 cigarettes a day since age 18, quit at age 31
- Does not drink any alcohol

Medications

- fluticasone propionate/salmeterol (Seretide) pMDI 250/25 micrograms, two inhalations twice daily starting 12 months ago
- salbutamol (Ventolin) pMDI 100 micrograms, 1–2 inhalations as required, repeated up to 4-hourly if needed

CASE STUDY 2: Merindah presents to the practice, as she continues to experience poor symptom control

- 41 years old
- Spirometry-confirmed asthma at age 29 years

Current history

- Daytime symptoms most days for previous 3 months
- Night waking more than once a week
- Needs salbutamol at least once a day
- Admission to ICU in the last year
- Confirmed good inhaler technique, adherence, self-management and knowledge of her asthma in a previous visit
- Follows her asthma action plan

Past medical history

- Diabetes, HbA_{1c} 7.9%
- GORD

Social

- Never smoked, drinks alcohol occasionally
- BMI > 32 kg/m^2

Medications

- budesonide/formoterol (Symbicort) Turbuhaler 400/12 micrograms, two inhalations twice daily
- salbutamol (Asmol) pMDI 100 micrograms, 1–2 inhalations as required, repeated up to 4-hourly if needed
- omeprazole EC (Acimax) 20 mg, 1 tablet daily
- metformin (Diabex) 1000 mg daily

Learning outcomes

1. Identify patients with difficult-to-treat asthma.
2. Assess asthma control, including symptom control and future risk of adverse outcomes, in patients with difficult-to-treat asthma.
3. Assess and manage factors that contribute to poor asthma control, including poor inhaler technique, poor adherence, comorbidities and triggers.
4. Identify patient characteristics for severe, high-risk and persistently difficult-to-treat asthma which may benefit from timely referral to a specialist.
5. Describe the role of biologic therapies in severe asthma and the rationale for their use.

ICS/LABA = inhaled corticosteroid/long-acting beta$_2$ agonist; OCS = oral corticosteroids; pMDI = pressurised metered-dose inhaler; GORD = gastro-oesophageal reflux disease

Figure 1: Systematic assessment of a patient with difficult-to-treat asthma

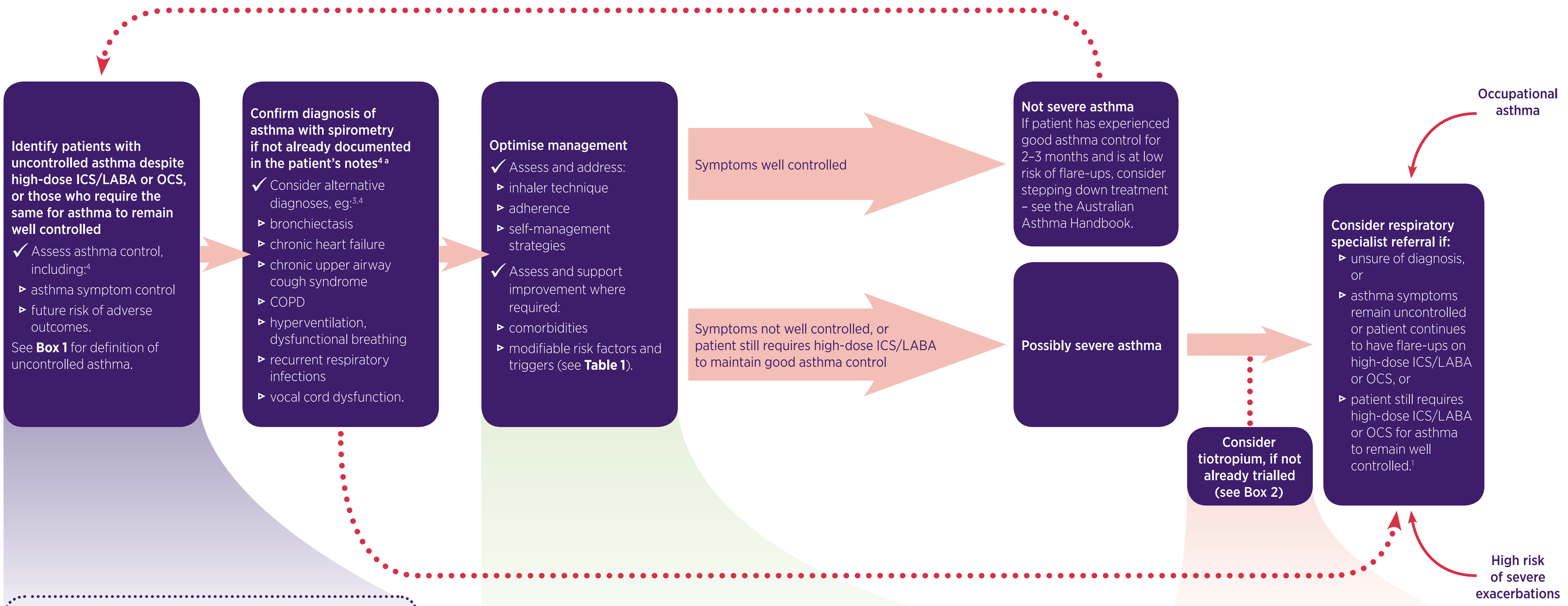

Box 1. What is uncontrolled asthma?

Uncontrolled asthma is defined as at least one of the following:[2]

1. Poor symptom control: in the last 4 weeks has the patient had at least one of the following:
   - daytime asthma symptoms more than twice per week?
   - any night waking due to asthma?
   - reliever needed for symptoms more than twice per week?
   - any activity limitation due to asthma?
2. Frequent severe exacerbations: two or more courses of OCS (> 3 days each) in the previous year
3. Serious exacerbations: at least one hospitalisation, ICU stay or episode of mechanical ventilation in the previous year
4. Airflow limitation: FEV_1 < 80% predicted (after appropriate bronchodilator withheld and with reduced FEV_1/FVC).

Uncontrolled asthma is also defined as controlled asthma that worsens on tapering high doses of ICS, OCS (or biologics).[2]

TABLE 1 **Modifiable factors that may contribute to poor symptom control**

Modifying the comorbidities in **bold** can particularly improve asthma control.

Medicines and related	Exposures	Comorbidities
▹ High SABA use<br>▹ Incorrect inhaler technique<br>▹ Medicines that may exacerbate asthma<br>▹ Poor adherence with preventer therapy	▹ Allergen exposure in sensitised patients (house dust mite, cat, mould, cockroach)<br>▹ Confirmed food allergy<br>▹ Indoor or outdoor air pollution, extreme weather<br>▹ Occupational exposure to allergens or irritants<br>▹ Respiratory viruses<br>▹ **Smoking or environmental tobacco smoke**, biomass fuel exposure	▹ Allergic bronchopulmonary aspergillosis<br>▹ Anxiety, depression<br>▹ COPD<br>▹ Bronchiectasis<br>▹ GORD<br>▹ **Obesity**<br>▹ Rhinosinusitis ± nasal polyposis<br>▹ Vocal cord dysfunction<br>▹ Pregnancy

Box 2. Tiotropium mist inhaler (Spiriva Respimat) for moderate to severe asthma

Available on the PBS general schedule as an add-on for adults with moderate to severe asthma[5] who:

- are on ICS ≥ 800 micrograms budesonide or equivalent per day plus a LABA, and
- who have had one or more severe asthma exacerbations in the previous year.[6]

Tiotropium is a LAMA that inhibits M3 receptors in the airways, resulting in relaxation of the airway smooth muscle.[7]

Compared to patients with severe asthma using ICS/LABA alone, a recent Cochrane review has found that adding tiotropium resulted in fewer exacerbations requiring OCS and is likely to have benefits on lung function and asthma control.[6]

Tiotropium should be stopped if no clinical benefit is seen.

a. Diagnosis is confirmed by compatible history, objective demonstration of variable expiratory airway obstruction using change in FEV_1, either spontaneously over time, before and after bronchodilator, or in response to a bronchial provocation agent (when baseline FEV_1 is normal).[5]

COPD = chronic obstructive pulmonary disease; FEV_1= forced expiratory volume in 1 second; FVC = forced vital capacity; SABA = short-acting $beta_2$ agonist; LAMA = long-acting muscarinic antagonist

Collaborate with respiratory specialists for patient-centred care

- Consider referral for patients with severe or persistently difficult-to-treat asthma to improve quality of life and allow timely access to specialist treatment options available for this patient population.[1]
- To be eligible for PBS-subsidised biologic therapy, patients must be under the care of a specialist experienced in the management of severe asthma for at least 12 months.[8,9]

Phenotyping and targeted treatments

- The respiratory specialist assesses and tailors treatment for patients with severe asthma based on inflammatory phenotypes.
- Three recognised clinical and inflammatory phenotypes are eosinophilic, allergic and non-eosinophilic asthma, and targeted treatments are now available for patients with severe asthma and an allergic or eosinophilic phenotype.[1,10]
- After initiation of biologic therapy by a specialist, the primary care prescriber facilitates the ongoing treatment in collaboration with the specialist.

TABLE 2

Laboratory markers and treatment associated with pathobiological characteristics of severe asthma in patients using high-dose ICS, according to inflammatory phenotype[1,10]

	ALLERGIC	EOSINOPHILIC AIRWAY INFLAMMATION	NEUTROPHILIC AIRWAY INFLAMMATION	MIXED INFLAMMATION	PAUCIGRANULOCYTIC ASTHMA
**Markers in patients using high-dose ICS**	Total serum IgE	Blood eosinophil count ≥ 0.3 cells x10^9/L, FeNO ≥ 20 ppb, sputum eosinophils ≥ 2%, low neutrophil percentage	≥ 40–60% polymorphonuclear neutrophils in sputum, low eosinophil percentage	High type 2 (eosinophil) and neutrophilic markers	No elevation of type 2 (eosinophilic) markers and ≤ 40–60% sputum polymorphonuclear neutrophils
**Treatment**	Omalizumab ± OCS	Mepolizumab ± OCS	LABA, LAMA, ?macrolide[b]		

b. Macrolides are not currently approved in Australia for long-term management of asthma

TABLE 3

PBS-subsidised biologic therapy

	OMALIZUMAB (XOLAIR)	MEPOLIZUMAB (NUCALA)
**PBS indication[c]**	Uncontrolled severe allergic asthma	Uncontrolled severe eosinophilic asthma
**Mechanism of action**	Monoclonal antibody that selectively binds to human IgE and limits the availability of mediators involved in the allergic cascade[11]	Monoclonal antibody directed against human IL-5, the major cytokine responsible for the growth and differentiation, recruitment, activation and survival of eosinophils[12]
**Benefits**	▸ Reduced asthma exacerbations<br>▸ Reduced need for ICS<br>▸ Improvement in asthma symptom control<br>▸ Improvement in quality of life[4]	▸ Reduced asthma exacerbations<br>▸ Oral-steroid sparing effect<br>▸ Improvement in quality of life[13–15]
**Common side effects**	▸ Injection-site reactions<br>▸ Anaphylaxis	▸ Injection-site reactions<br>▸ Headache
**Administration**	▸ Subcutaneous injection every 2–4 weeks[11]<br>▸ The patient should remain for 2 hours under direct staff observation after the first three doses; and 30 minutes for subsequent doses[16]	▸ Subcutaneous injection every 4 weeks[12]<br>▸ The patient should remain for 1 hour under direct staff observation after the first dose; and 30 minutes for subsequent doses[17]

c. Authority Required: Refer to PBS Schedule for full authority information. Information current at 9 January 2018.

ppb = parts per billion; IgE = immunoglobulin E; FeNO = fractional exhaled nitric oxide; IL-5 = interleukin 5

EXPERT REVIEWER

Professor Helen Reddel
Respiratory Physician
Professor, Woolcock Institute of Medical Research, University of Sydney, and Royal Prince Alfred Hospital.

References available online at: **nps.org.au/asthma-card-refs**

This program is joint-funded by AstraZeneca Pty Ltd and Novartis Pharmaceuticals Australia Pty Ltd and managed by VentureWise, a wholly owned commercial subsidiary of NPS MedicineWise. The content has been designed, developed and delivered by NPS MedicineWise with complete independence and editorial control and is based on best practice guidelines.

VENTUREWISE
Part of the MedicineWise Group

NPS MEDICINEWISE PROFESSIONAL

Level 7/418A Elizabeth Street Surry Hills NSW 2010
PO Box 1147 Strawberry Hills NSW 2012
02 8217 8700 02 9211 7578 info@nps.org.au

www.nps.org.au
www.venturewise.com.au

Independent. Not-for-profit. Evidence-based.

© 2018 NPS MedicineWise and VentureWise Pty Ltd. Reasonable care is taken to provide accurate information at the time of creation. This information is not intended as a substitute for medical advice and should not be exclusively relied on to manage or diagnose a medical condition. NPS MedicineWise and VentureWise disclaim all liability (including for negligence) for any loss, damage or injury resulting from reliance on or use of this information. January 2018.

NPS1936b
