# Supplementary material for: Difficult-to-treat and severe asthma in general practice: delivery and evaluation of an educational program
Source: BMC Fam Pract. 2019 Jul 13;20:99. doi: 10.1186/s12875-019-0991-y (PMC6626400; doi:10.1186/s12875-019-0991-y)
Supplement: Supplementary file 2 — The RPT questionnaire provided to GPs at the end of the educational visit. (PDF 385 kb) [file 12875_2019_991_MOESM2_ESM.pdf]

NPS MEDICINEWISE

CELEBRATING 20 YEARS 1998–2018

GP Survey

Managing people with difficult-to-treat asthma: a systematic approach

Please take 5-10 minutes to complete this survey. Your responses will assist us in evaluating our program and understanding GP knowledge, attitudes, and practice related to difficult-to-treat asthma.

Participation in this survey is voluntary and will not have any impact on your relationship with NPS MedicineWise. By completing the survey, you are providing implied consent for your responses to be used for evaluation purposes. This may include evaluation of this and future programs delivered by NPS MedicineWise.

Your responses are confidential and will be aggregated for the purposes of reporting to program funders and for publications; no individual responses will be identifiable. This project has received ethics approval from the RACGP National Research and Evaluation Ethics Committee (Reference number: 17-018).

If you have any concerns about how the study is being conducted or the consent process please contact the committee on **ethics@racgp.org.au** or **03 8699 0497.**

If you have any other questions, please contact Kirsten Sterling by email **ksterling@nps.org.au** or phone on **02 8217 8773**.

A number of questions ask you to provide an answer for two different time periods. The first period (NOW) refers to your current attitudes and intended practice. The second period (BEFORE) refers to your attitudes and practice before participating in the difficult-to-treat asthma program.

Please mark your answers by crossing the box as instructed in the questions. ☒

Please place your completed survey in the envelope provided at your session, or return by fax **02 9211 7578** or email **ksterling@nps.org.au**

If you prefer to complete this survey online, please visit **nps.org.au/asthmasurvey**

1. The visit discussed conditions including uncontrolled asthma, difficult-to-treat asthma, and severe asthma. We would like to understand how you use these terms in your practice.

For each statement about asthma below, please select which condition(s) the statement relates to.
(You may select more than one answer per statement if it is relevant to more than one condition).

	Uncontrolled asthma	Difficult-to-treat asthma	Severe asthma
Asthma that includes at least one of the following: poor symptom control, frequent severe exacerbations, serious exacerbations and/or airflow limitation	☐	☐	☐
Asthma that is uncontrolled despite high-dose ICS/LABA and/or oral corticosteroids, or that requires such treatment to remain well controlled	☐	☐	☐
Asthma that is uncontrolled despite high-dose ICS/LABA and/or oral corticosteroids and does not improve following appropriate diagnosis, optimisation of inhaled treatment and/or treatment of confounders	☐	☐	☐

6. Please rate the degree to which your learning needs were met by this educational visit:

☐ Entirely met ☐ Partially met ☐ Not met

7. Please rate the degree to which this educational visit was relevant to your own practice:

☐ Entirely relevant ☐ Partially relevant ☐ Not relevant

8. Please rate your overall satisfaction with this educational visit.

☐ Entirely satisfied ☐ Partially satisfied ☐ Not satisfied

9. Please rate the suitability of the mode of delivery of this educational visit.

☐ Entirely suitable ☐ Partially suitable ☐ Not suitable

10. In your opinion, was the information discussed with you during the educational visit?

☐ Too challenging ☐ Just right ☐ Too basic

About you and your practice

11. Are you?

☐ Male ☐ Female

12. How many years have you practiced as a GP?

☐☐

13. What is the postcode of your main practice?

☐☐☐☐

14. Approximately how many patients would you see in a usual week?

☐☐☐

15. Each week, approximately how many patients would you see with a diagnosis of asthma?

☐☐☐

16. Please provide any other feedback or suggestions for improvement of this activity:

....

....

....

P: 02 8217 8700
F: 02 9211 7578
info@nps.org.au
nps.org.au

Independent, not-for-profit, evidence-based, NPS MedicineWise enables better decisions about medicines, medical tests and other health technologies. We receive funding from the Australian Government Department of Health.

© 2018 NPS MedicineWise NPS2007

2. When a patient with poorly controlled asthma presents to your practice, what factors would you assess in managing their condition?

Please indicate the responses most consistent with your intended practice NOW and BEFORE participating in an educational activity with an NPS Medicinewise Clinical Services Specialist.

(Select all that apply)

	NOW	BEFORE
Inhaler technique		
Medicine adherence		
Comorbidities		
Self-management strategies		
Trigger factors		
Changes in lifestyle (exercise, weight, etc)		
Confirmation of diagnosis with spirometry		
Adequate trial of preventer medicines		
Use of a written action plan		
Risk of future adverse outcomes		
Other (Please specify): ________________		

3. In which situations would you consider referring a patient with difficult-to-treat or severe asthma to a respiratory specialist?

Please indicate the responses most consistent with your intended practice NOW and BEFORE participating in an educational activity with an NPS MedicineWise Clinical Services Specialist.

(Select all that apply)

	NOW	BEFORE
The patient requires high-dose inhaled corticosteroids to maintain asthma control despite correct inhaler technique and good adherence		
I have been treating the patient for 12 months or more with little to no improvement in symptoms		
The patient is at high risk of future adverse asthma-related outcomes		
I have addressed comorbidities I can treat and have not seen an improvement in asthma control		
I am unsure of the asthma diagnosis (eg, patients with features of both asthma and COPD)		
I suspect the patient has occupational asthma		
I believe the patient may have severe asthma and may be a candidate for treatment with a biologic therapy		
Other (Please specify): ________________		

4. Please rate your level of agreement with the following statements

Please indicate the responses most consistent with your level of agreement NOW and BEFORE participating in an educational activity with an NPS MedicineWise Clinical Services Specialist.

(For each row, please select one answer for NOW and one for BEFORE the educational visit)

	NOW					BEFORE				
	Strongly agree	Agree	Neutral	Disagree	Strongly disagree	Strongly agree	Agree	Neutral	Disagree	Strongly disagree
I would feel confident managing a patient I have identified as having difficult-to-treat asthma										
I understand that different patients with asthma may have different phenotypic characteristics that can respond differently to standard therapy										
I am aware of available biologic therapies that respiratory specialists can consider prescribing to my patients with severe asthma										
I understand the PBS requirements for a patient to be prescribed a biologic therapy for severe asthma										
I have a good understanding of which patients would benefit from timely referral to a respiratory specialist										
I have a good understanding of the role of GPs and specialists in collaboratively managing a patient with severe asthma who requires biologic therapy										

Learning outcomes and your practice

5. As a result of participation in this educational visit, the below learning outcomes have been:

	Entirely met	Partially met	Not met
Identify patients with difficult-to-treat asthma			
Assess asthma control, including symptom control and future risk of adverse outcomes, in patients with difficult-to-treat asthma			
Assess and manage factors that contribute to poor asthma control, including poor inhaler technique, poor adherence, comorbidities and triggers			
Identify patient characteristics for severe, high-risk and uncontrolled asthma who may benefit from timely referral to a specialist			
Describe the role of biologic therapies in severe asthma and the rationale for their use			
